# Palladium(ii)-Acetylacetonato Complexes with Mesoionic Carbenes: Synthesis, Structures and Their Application in the Suzuki-Miyaura Cross Coupling Reaction

**DOI:** 10.3390/molecules21111561

**Published:** 2016-11-17

**Authors:** Lara Hettmanczyk, Bianca Schmid, Stephan Hohloch, Biprajit Sarkar

**Affiliations:** Institut für Chemie und Biochemie, Anorganische Chemie, Freie Universität Berlin, Fabeckstraße 34-36, Berlin D-14195, Germany; Lara.Hettmanczyk@fu-berlin.de (L.H.); bianca.schmid@chem.tu-berlin.de (B.S.); Stephan.Hohloch@web.de (S.H.)

**Keywords:** mesoionic carbenes, triazolylidenes, palladium, Suzuki-Miyaura cross coupling, click chemistry, abnormal carbenes

## Abstract

A series of novel palladium(ii) acetylacetonato complexes bearing mesoionic carbenes (MICs) have been synthesized and characterized. The synthesis of the complexes of type (MIC)Pd(acac)I (MIC = 1-mesityl-3-methyl-4-phenyl-1,2,3-triazol-5-ylidene (**1**), 1,4-(2,4,6-methyl)-phenyl-3-methyl-1,2,3-triazol-5-ylidene (**2**), 1,4-(2,6-diisopropyl)-phenyl-3-methyl-1,2,3-triazol-5-ylidene (**3**); acac = acetylacetonato) via direct metalation starting from the corresponding triazolium iodides and palladium(ii) acetylacetonate is described herein. All complexes were characterized by ^1^H- and ^13^C-NMR spectroscopy and high resolution mass spectrometry. Additionally, two of the complexes were characterized by single crystal X-ray crystallography confirming a square-planar coordination geometry of the palladium(ii) center. A delocalized bonding situation was observed within the triazolylidene rings as well as for the acac ligand respectively. Complex **2** was found to be an efficient pre-catalyst for the Suzuki-Miyaura cross coupling reaction between aryl-bromides or -chlorides with phenylboronic acid.

## 1. Introduction

*N*-heterocyclic carbenes (NHCs) are a very attractive class of compounds. Because of their easy accessibility and their high complex stability, these ligands have been used extensively in organometallic chemistry in the last decades [1,2,3,4,5,6]. They are mainly applicable in homogeneous catalysis [7,8] but also in other fields of chemistry such as material science [9,10], bioorganometallic chemistry [10,11,12] or metallosupramolecular chemistry [13,14,15]. A special kind of NHCs are mesoionic carbene (MIC), which have received high popularity in recent years. Apart from imidazol-5-ylidenes, 1,2,3-triazol-5-ylidenes are the most noted MICs found in the literature [16,17,18,19,20,21,22]. Their precursors, the 1,2,3-triazoles, are easily accessible via the copper(i) catalyzed azide-alkyne cycloaddition (CuAAC) reaction [23,24,25,26,27]; subsequent alkylation results in the corresponding 1,2,3-triazolium salts, which can be converted into mesoionic carbenes [6,22,28]. Metal complexes of 1,2,3-triazol-5-ylidenes are used as catalysts in a variety of catalytic transformations [16,17,18,19,20,21]. For example, copper(i) complexes have been found to be potent catalysts for the “click” reaction [29,30,31,32,33,34], while cyclization reactions can be performed using gold(i) 1,2,3-triazol-5-ylidene complexes as catalysts [14,28,35,36,37,38,39,40,41]. Iridium(iii) complexes were shown to be efficient catalysts in water oxidation reactions [9,42,43,44,45] as well as other oxidation reactions and transfer hydrogenations [46,47,48,49,50,51], which are also catalyzed by ruthenium(ii) and osmium(ii) complexes [46,47,49,50,52,53,54,55,56,57,58]. Furthermore, 1,2,3-triazol-5-ylidenes and their metal complexes have also been investigated for their photochemical [59,60,61,62,63,64] and redox properties [60,65]. 

**Figure 1:**
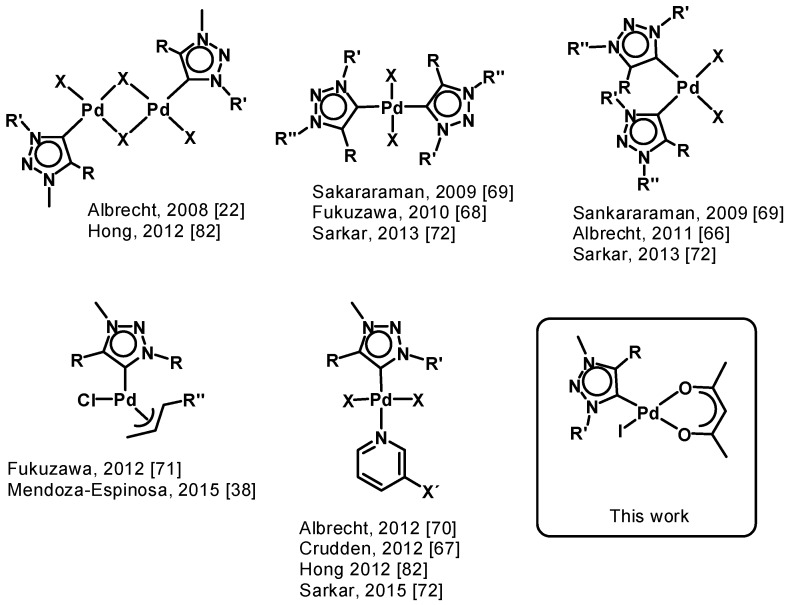
Overview of selected palladium(ii) complexes with 1,2,3-triazol-5-ylidene ligands.

Especially palladium(ii) 1,2,3-triazol-5-ylidene complexes obtained a lot of attention since the discovery of 1,2,3-triazol-5-ylidene complexes [22]. Various types of palladium(ii) complexes show diversities in the coordination fashion and have different additional ligands (Figure 1) [22,38,65,66,67,68,69,70,71,72,73,74,75,76,77,78,79,80,81,82]. Almost all palladium(ii) complexes were used as catalysts in a variety of cross coupling reactions. Mainly, they are used as pre-catalysts in reactions like the Suzuki-Miyaura cross coupling reaction [68,69,70,71,72,73,74,75,80,81,82,83] or α-arylation and α-methylation reactions [38,75,80].

We have been interested in the development of a novel coordination motif of palladium(ii) 1,2,3-triazol-5-ylidenes complexes, in which κ^2^-acetylacetonate is coordinated as additional chelating ligand to the palladium(ii) center. For normal *N*-heterocyclic carbenes (NHC), these kind of palladium(ii) complexes, but with chlorido instead of iodido as ligand, were first reported in 2005 [84]. These NHC complexes showed high catalytic activity in the Buchwald–Hartwig amination and in the arylation of α-ketones [85,86].

Herein we report on the synthesis of a series of novel palladium(ii) acetylacetonato complexes bearing mesoionic carbenes. The complexes have been characterized by ^1^H- and ^13^C-NMR spectroscopy, mass spectrometry and single crystal X-ray crystallography. Their catalytic activity in the Suzuki-Miyaura cross coupling reaction are presented as well.

## 2. Results and Discussion

### 2.1. Synthesis and Characterization of the Palladium(ii) Complexes

**Scheme 1:**
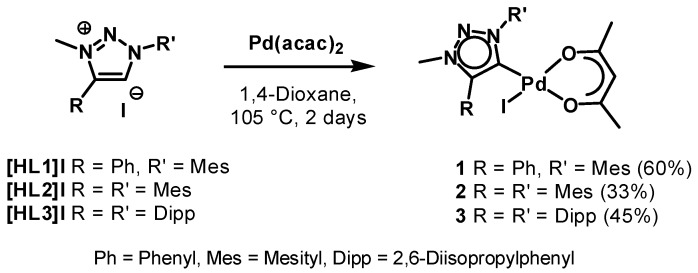
Synthesis of the palladium(ii) MIC complexes.

The triazolium salts **[HL1]I** [70,87], **[HL2]I** [68] and **[HL3]I** [32] were synthesized according to procedures reported in the literature. The complexes **1**–**3** were synthesized in a one-pot reaction starting from the corresponding triazolium salts via direct metalation by use of [Pd(acac)_2_] under inert conditions (Scheme 1). The route used here is similar as for the preparation of related nNHC complexes [85], but for the triazolylidene complexes the iodides of the triazolium salts were used because of synthetic ease. One of the acac ligands on [Pd(acac)_2_] acts as an internal base for the deprotonation of the triazolium salts. The coordinated iodides on the palladium centers originate from the corresponding triazolium salts (Scheme 1). The identity and purity of the compounds were unambiguously proven by mass spectrometry, ^1^H- and ^13^C-NMR spectroscopy. All complexes were isolated as yellow solids after precipitation or as yellow crystalline solids after recrystallization from a mixture of dichloromethane and hexane. In contrast to the achieved moderate to good yields of these MIC complexes, the yields obtained for the nNHC complexes have been nearly quantitative [85], even under aerobic conditions [86]. The reason for this might be the higher acidity of the imidazolium-*2H* [88,89,90] in comparison with the acidity of the triazolium-5*H* [19,91]. While the solubility of complexes **1**–**3** is very high in THF, diethyl ether, toluene or chlorinated solvents, the complexes were only moderately to sparingly soluble in hexane or pentane.

The first indication for the formation of the palladium(ii) MIC complexes in solution was the disappearance of the triazolium-*5H* in the ^1^H-NMR spectra and the appearance of the single proton of the bound acac ligand at 5.14, 5.12 and 5.13 ppm for **1**, **2** and **3** respectively. Further proof was given by ^13^C-NMR spectroscopy based on the signals of the carbene carbons at 151.1 and 150.2 ppm for **2** and **3** respectively. Unfortunately, the signal corresponding to the carbene carbon for **1** was not resolved in its ^13^C-NMR spectrum. Two signals of the quaternary carbons of the bound acac ligand was observable for each complex in the range of 183–187 ppm (see Figures S1–S3). For the methyl-group of the acac ligand two separate signals could be observed in the ^1^H-NMR as well as in the ^13^C-NMR spectra. Additional characterization of the complexes by ESI-MS showed molecular peaks corresponding to the molecular mass with loss of the iodide (see ESI).

### 2.2. Structural Characterization

**Figure 2:**
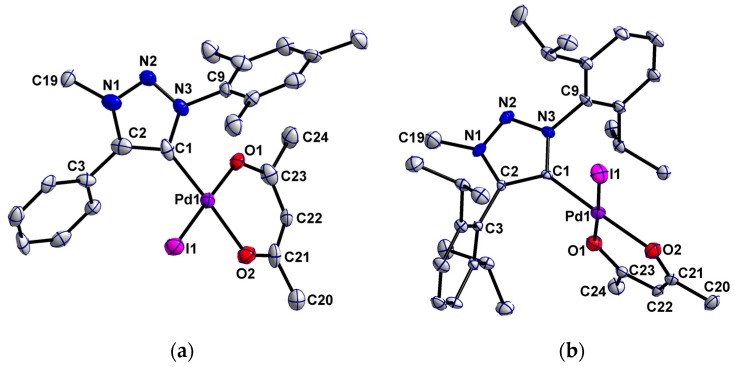
Perspective view of complexes **1** (**a**) and **3** (**b**). Ellipsoids are drawn at the 50% probability level. Hydrogen atoms have been omitted for clarity.

The molecular structures of palladium(ii) complexes **1** and **3** in crystal could be confirmed by single crystal X-ray crystallography (Figure 2). Single crystals were grown by slow diffusion of hexane onto a concentrated solution in dichloromethane. Crystallographic details for these complexes are given in Table S1. Important bond length and angles are depicted in Table 1.

Complex **1** crystallized as yellow needles in the orthorhombic space group *Pcba*, while complex **3** crystallized as yellow plates in the monoclinic space group P2_1_/*n* (see Table S1). The C–C, C–N and N–N bond lengths and the corresponding angles within the five-membered triazolylidene rings indicate a delocalized bonding situation in those rings for both complexes (Table 1). The aryl-substituents are twisted with respect to the triazolylidene ring. In complex **1** the dihedral angles between the aryl-substituents and the triazolylidene ring are 46.7° and 47.5°. The Dipp-substituents in complex **3** are almost perpendicular to the triazolyldiene rings with dihedral angles of 86.0° and 71.1°. The large twists observed in the aforementioned case is likely due to the higher steric demand of the Dipp substituents (Table 1). Within the acac ligand, the bond length and angles are also indicative of a delocalized bonding situation.

Both palladium(ii) MIC complexes display an almost square-planar coordination geometry, in which the angles between palladium and the different ligands are in between 88.7(2)° and 91.9(3)°. The square-planar geometry is typical for palladium(ii) centers with a d^8^ electronic configuration. The related nNHC complex showed a slightly higher deviation from the perfect square-planar geometry [84]. In complex **1** the triazolylidene ring is twisted about 57.1° with respect to the acac-Pd plane, while in complex **3** the triazolylidene ring is rotated about 82.7° resulting from the higher steric demand of the Dipp substituents (Table 1).

The distances between the palladium atoms and the carbene carbon atoms are almost similar in both complexes with 1.98 (1) Å and 1.957 (5) Å respectively, which is in accordance with other palladium(ii) triazolylidene complexes [65,75,80,92]. The Pd-C bond length of 1.969 (2) Å for the related nNHC complex lies in between the Pd-C distances of the MIC complexes reported here [84]. The Pd-I distances of 2.561 (1) Å and 2.560 (1) Å are a bit lower compared to Pd-I bond distances in other palladium(ii) MIC complexes [65,75,80,92]. While the acac ligand in **1** is unsymmetrically bound to the Pd center (2.027 (7) Å and 2.060 (7) Å) in **3** the bonding situation was found to be more symmetric with Pd-O1 and Pd-O2 being 2.054 (4) Å and 2.046 (4) Å respectively. For the related nNHC complex the Pd-O bond length are shorter with 2.044 (1) Å and 2.036 (2) Å compared to the MIC complexes [84]. 

### 2.3. Catalysis

Palladium(ii) NHC complexes in general are known to be effective catalysts in various transformations and therefore they play a central role in organic chemistry [3,7,93,94]. Different bonds such as C–X (X = halide) can be activated and new C–C or C–N bonds can be built. Also, many examples for catalytically active palladium(ii) MIC complexes in cross coupling reactions are known in the literature. The main application was reported in Suzuki–Miyaura coupling reactions [68,69,70,71,72,73,74,75,80,81,83], Mizoroki-Heck reactions [67] or Hiyama coupling reactions [77,78]. Other reactions like α-arylation or α-methylation [38,75,80] and the Buchwald–Hartwig reaction [79] were presented as well.

**Scheme 2:**
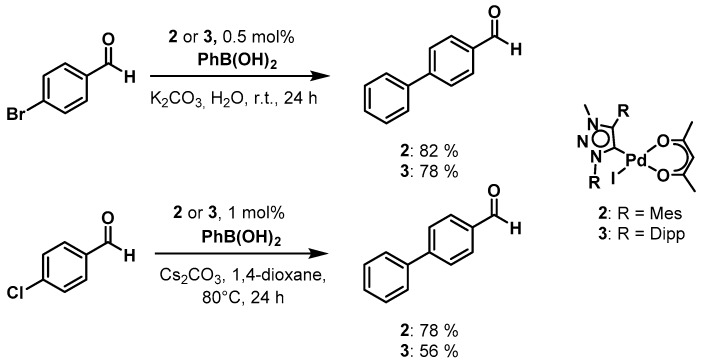
Palladium(ii) mesoionic carbene (MIC) complexes **2** and **3** as pre-catalysts in the Suzuki-Miyaura cross coupling reaction.

To gain an insight into the catalytic activity of our novel palladium(ii) MIC complexes we have chosen the Suzuki-Miyaura cross coupling and the Buchwald-Hartwig amination reactions as examples and complexes **2** and **3** as pre-catalysts. Both complexes show good to excellent activities in the Suzuki-Miyaura cross-coupling reaction between aryl bromides and chlorides with phenylboronic acid (Scheme 2).

**Scheme 3:**
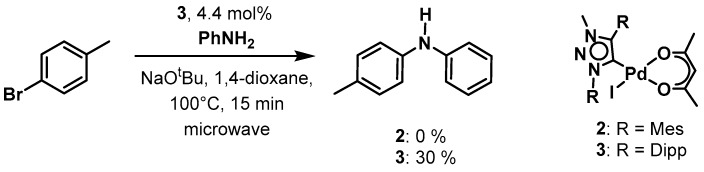
Palladium(ii) MIC complex **3** as pre-catalyst in the Buchwald-Hartwig amination.

For 4-bromobenzaldehyde, the reaction could be performed in the environment-friendly solvent water at room temperature under air, using potassium carbonate as base. With a catalyst loading of only 0.5 mol % of **2** a conversion of 82% could be obtained within 24 h. For complex **3**, the conversion was fairly similar at 78%. In the case of 4-chlororbenzaldehyde, which tends to be less easy to activate, the reaction conditions had to be changed. Therefore, we used 1,4-dioxane as the solvent under an Ar atmosphere, cesium carbonate as a base and the reaction temperature was increased to 80°. Applying these conditions, we were able to achieve a conversion of 78% within 24 h with catalyst loadings of 1 mol % of **2**. For **3**, the conversion was 56% under the same conditions. Additionally, we investigated the Buchwald-Hartwig amination reaction with both **2** and **3** as pre-catalysts under microwave irradiation conditions. This was necessary as the Buchwald-Hartwig amination is intrinsically a more difficult reaction to perform in comparison to the Suzuki-Miyaura cross-coupling reaction. Unfortunately, with complex **2**, no conversion was observed under the used conditions. With complex **3**, however, we were able to observe 30% conversions to product by using 4.4% catalyst loading under only 15 min of microwave irradiation (Scheme 3). The investigation of these two classes of catalytic reactions shows that the substituents on the triazolylidene ligands do have a direct or an indirect influence on the catalytic activities of the resulting palladium complexes. In the Suzuki-Miyaura cross-coupling reaction, the activity of the complexes presented here seems to be better than those of the corresponding PEPPSI complexes containing triazolylidene ligands, in which case a catalyst loading of 2.5 mol % (in comparison to 0.5 mol %) was necessary with similar substrates [80]. These results indicate a positive effect of the use of the acac containing complexes for these kinds of catalysis. 

## 3. Materials and Methods 

### 3.1. General Procedures, Materials and Instrumentations

The triazolium salts **[HL1]I** [70,87], **[HL2]I** [68] and **[HL3]I** [32] were prepared as described previously in the literature. Commercially available chemicals were used as purchased, unless otherwise noted. The solvents used for synthesis and catalysis were dried and distilled under inert gas and degassed by common techniques prior to use, unless otherwise noticed. ^1^H- and ^13^C-NMR spectra were recorded on a Jeol ECS 400 or Jeol ECZ 400R spectrometer (Joel, Munich, Germany) using the chemical shift of the solvent as an internal standard. Multiplets are reported as follows: singlet (s), duplet (d), triplet (t) quartet (q), quintet (quint), and combinations thereof. Mass spectrometry was performed on an Agilent 6210 ESI-TOF (Agilent, Walbronn, Germany). 

### 3.2. X-ray Crystallography

X-Ray data were collected on a Bruker Smart AXS diffractometer (Bruker, Karlsruhe, Germany). Data were collected at 140(2) K (see Table S1) using graphite-monochromated Mo Kα radiation (λ_α_ = 0.71073 Å). The strategy for the data collection was evaluated by using the Smart software (Bruker, AXS Inc., Madison, WI, USA). The data were collected by the standard ‘ωscan techniques’ and were scaled and reduced using Saint+ and SADABS software. The structures were solved by direct methods using SHELXS-97 or SHELXS_2014/7 and refined by full matrix least-squares, refining on F^2^. Non-hydrogen atoms were refined anisotropically [95,96,97,98,99,100,101]. If it is noted, bond length and angles were measured with Diamond Crystal Version 4.0.2 (Crystal Impact GbR, Bonn, Germany) and Molecular Structure Visualization Version 3.1 (Crystal Impact GbR). CCDC 965893 and 1015507 contains the supplementary crystallographic data for this paper. These data can be obtained free of charge via http://www.ccdc.cam.ac.uk/conts/retrieving.html (or from the CCDC, 12 Union Road, Cambridge CB2 1EZ, UK; Fax: +44 1223 336033; E-mail: deposit@ccdc.cam.ac.uk)

### 3.3. Synthesis

#### 3.3.1. General Procedure for the Preparation of the Palladium(ii) 1,2,3-Triazolylidene Complexes **1**–**3**

The corresponding triazolium salt **[HL1]I**, **[HL2]I** or **[HL3]I** (0.2 mmol, 1 eq.) and palladium(ii) acetylacetonate (60.9 mg, 0.2 mmol, 1 eq.) were dissolved in absolute 1,4-dioxane (3 mL) and stirred for 48 h at 105 °C. The reaction mixture was filtered over Celite and volatiles were removed under vacuum. The residue was re-resolved in dichloromethane (2 mL) and precipitated with diethyl ether (50 mL) to remove unreacted triazolium salt. The filtrate was reduced and precipitated again with hexane (50 mL) to remove the unreacted Pd(acac)_2_, which is soluble in hexane. The yellow precipitate was collected by filtration and washed with hexane. Recrystallization from a mixture of dichloromethane and hexane (1:4) gave the complexes as crystalline yellow solids. Single crystals suitable for X-ray diffraction analysis were obtained by slow diffusion of hexane onto a concentrated solution of the complex in dichloromethane.

*Palladium(ii) complex* **1**: The product was obtained as yellow solid in a yield of 60% (73.1 mg, 0.12 mmol). ^1^H-NMR (401 MHz, CDCl_3_, 23 °C) δ = 7.96–7.91 (m, 2H, Ar-*H*), 7.61–7.50 (m, 3H, Ar-*H*), 7.04–6.98 (m, 2H, Ar-*H*), 5.14 (s, 1H, C*H* of acac), 4.11 (s, 3H, NC*H*_3_), 2.37 (s, 3H, C*H*_3_), 2.29 (s, 3H, C*H*_3_), 2.16 (s, 3H, C*H*_3_), 1.85 (s, 3H, C*H*_3_ of acac), 1.76 (s, 3H, C*H*_3_ of acac) ppm. ^13^C-NMR (101 MHz, CDCl_3_, 24 °C) δ = 186.6, 183.6 (*C*O, acac), 145.6, 141.4, 140.5, 135.6, 135.3, 130.8, 130.1, 129.0, 127.3 (all Aryl-*C*), 99.1 (*C*H, acac), 37.8 (N*C*H_3_), 27.6, 26.3, 21.4 (all Alkyl-*C*) ppm. HRMS (ESI): calcd for [C_23_H_26_IN_3_O_2_Pd] [M − I]^+^ *m*/*z* 482.1060, found 482.1065.

*Palladium(ii) complex* **2**: The product was obtained as yellow solid in a yield of 33% (43.0 mg, 0.066 mmol). ^1^H-NMR (401 MHz, CDCl_3_, 22 °C) δ = 7.01 (s, 4H, Aryl-*H*), 5.12 (s, 1H, C*H* of acac), 3.80 (s, 3H, NC*H*_3_), 2.37 (*p-*d, 6H, C*H*_3_), 2.27–2.19 (m, 12H, C*H*_3_), 1.81 (s, 3H, C*H*_3_ of acac), 1.76 (s, 3H, C*H*_3_ of acac) ppm. ^13^C-NMR (126 MHz, CDCl_3_, 21 °C) δ = 186.4, 183.2 (*C*O, acac), 151.1 (Carbene-*C*), 147.8, 144.5, 141.5, 140.5, 140.4, 139.1, 135.6, 135.5, 122.9 (all Aryl-*C*), 99.0 (*C*H, acac), 36.5 (N*C*H_3_), 29.8, 27.6, 26.2, 21.5, 21.4 (all Alkyl-*C*) ppm. HRMS (ESI): calcd for [C_26_H_32_IN_3_O_2_Pd] [M − I]^+^ *m/z* 524.1529, found 524.1518.

*Palladium(ii) complex* **3**: The product was obtained as yellow solid in a yield of 45% (66.2 mg, 0.09 mmol). ^1^H-NMR (400 MHz, CDCl_3_, 20 °C) δ = 7.53 (t, *J* = 7.8 Hz, 2H, Aryl-*H*), 7.34 (d, *J* = 7.8 Hz, 4H, Aryl-*H*), 5.13 (s, 1H, C*H* of acac), 3.78 (s, 3H, NC*H*_3_), 2.99 (hept, *J* = 6.9 Hz, 2H, C*H*), 2.92–2.82 (m, 2H, C*H*), 1.86 (s, 3H, C*H*_3_ of acac), 1.81 (s, 3H, C*H*_3_ of acac), 1.36–1.32 (m, 12H, C*H*_3_), 1.07 (dd, *J* = 10.8, 6.9 Hz, 12H, C*H*_3_) ppm. ^13^C-NMR (101 MHz, CDCl_3_, 22 °C) δ = 186.0, 183.5 (*C*O, acac), 150.2 (Carbene-*C*), 146.1, 143.5, 143.3, 136.1, 131.5, 131.3, 124.2, 124.2, 123.4 (all Aryl-*C*), 98.8 (*C*H, acac), 37.5 (N*C*H_3_), 30.9, 29.1, 27.7, 27.5, 26.0, 25.1, 24.9, 22.9 (all Alkyl-*C*) ppm. HRMS (ESI): calcd for [C_32_H_44_IN_3_O_2_Pd] [M − I]^+^ *m/z* 608.2468, found 608.2471.

#### 3.3.2. Suzuki-Miyaura Cross Coupling Reactions

The 4-bromobenzaldeyhde (92.5 mg, 0.5 mmol, 1 eq.), phenylboronic acid (73.2 mg, 0.6 mmol, 1.2 eq.) and potassium carbonate (103.9 mg, 0.75 mmol, 1.5 eq.) were mixed in the presence of water (1.5 mL) under air. Then complexes **2** or **3** (0.5 mol %) was added. The mixture was stirred at room temperature for 24 h. For the aryl-chloride, 4-chlorobenzaldeyhde (28.1.5 mg, 0.2 mmol, 1 eq.), phenylboronic acid (20.3 mg, 0.24 mmol, 1.2 eq.) and cesium carbonate (130.3 mg, 0.4 mmol, 2 eq.) were mixed in presence of absolute 1,4-dioxane (1 mL) under nitrogen atmosphere. After that, complexes **2** or **3** (1 mol %) was added. The mixture was stirred at 80 °C for 24 h. The crude reaction mixtures were poured into dichloromethane respectively and extracted with water five times. Finally, the combined aqueous layers were extracted one last time with dichloromethane. The combined organic layers were dried over sodium sulfate, filtered and evaporated to dryness. Afterwards, yields were determined via ^1^H-NMR spectroscopy with the help of the aldehyde protons.

#### 3.3.3. Buchwald-Hartwig Amination

4-Bromobenzene (34.2 mg, 0.2 mmol, 1 eq.), aniline (21.9 μL, 22.4 mg, 0.24 mmol, 1.2 eq.), NaOtBu (23.1 mg, 0.24 mmol, 1.2 eq.) and the complexes **2** or **3** (4.4 mol %) were mixed in the presence of absolute 1,4-dioxane (2 mL). The reaction mixture was stirred at 100 °C for 15 min in the microwave. The reaction mixture was cooled to room temperature, and the solvent was removed under vacuum. Afterwards, yields were determined via ^1^H-NMR spectroscopy with the help of the protons of the methyl-group of the product with 1,3,5-tribromobenzene as internal standard.

## 4. Conclusions 

In conclusion, we have presented here the first examples of three palladium(ii) MIC complexes of the type (MIC)Pd(acac)I. Additional to the MIC, the chelating bidentate ligand *κ*^2^-acetylacetonate is coordinated to the palladium(ii) center. The synthesis and characterization of all complexes by mass spectrometry, ^1^H- and ^13^C-NMR spectroscopy is described herein. The molecular structure in crystal was verified for two of the three palladium(ii) complexes by single crystal X-ray crystallography. Structural characterization revealed the expected square-planar coordination fashion of the palladium(ii) center. Due to the popular application of palladium(ii) MIC complexes as pre-catalysts in cross coupling reactions, we utilized one of our new complexes as a pre-catalyst in the Suzuki–Miyaura cross coupling reaction. First attempts showed that these kind of complexes are excellent pre-catalysts for the cross coupling of phenylboronic acid with an aryl-bromide as well as an aryl-chloride. The inclusion of the acac ligand on the Pd-MIC unit opens up new possibilities for the synthesis of novel palladium complexes with mesoionic carbene ligands. Changing the donor properties of the acac ligand is conceivable, as is its substitution with other chelating anionic ligands. Such new motifs might open up additional opportunities and perspectives in the chemistry of the metal complexes of MIC ligands.

## Figures and Tables

**Table 1:** Selected bond length (Å) and angles (°).

Atoms	1	3
C1-C2	1.37 (2)	1.387 (6)
C1-N3	1.36 (1)	1.376 (6)
C2-N1	1.37 (1)	1.354 (6)
N1-N2	1.32 (1)	1.304 (6)
N2-N3	1.32 (1)	1.351 (6)
Pd1-C1	1.98 (1)	1.957 (5)
Pd1-I1	2.561 (1)	2.560 (1)
Pd1-O1	2.027 (7)	2.054 (4)
Pd1-O2	2.060 (7)	2.046 (4)
O1-C23	1.27 (1)	1.264 (6)
O2-C21	1.27 (1)	1.281 (7)
C23-C22	1.39 (2)	1.398 (8)
C21-C22	1.42 (2)	1.379 (8)
C1-C2-N1	106 (1)	107.5 (4)
C2-C1-N3	103 (1)	103.3 (4)
C1-N3-N2	114.9 (8)	112.0 (4)
C2-N1-N2	112.5 (9)	111.9 (4)
N1-N2-N3	103.1 (8)	105.4 (4)
C23-C22-C21	127 (1)	127.0 (5)
O1-C23-C22	127 (1)	126.8 (5)
O2-C21-C22	125 (1)	127.0 (5)
C1-Pd1-I1	90.4 (3)	88.9 (1)
C1-Pd1-O1	89.1 (4)	88.7 (2)
C1-Pd1-O2	177.1 (4)	178.4 (2)
I1-Pd1-O1	175.3 (2)	177.4 (1)
I1-Pd1-O2	88.8 (2)	90.5 (1)
O1-Pd1-O2	91.9 (3)	91.9 (2)
trz-Ar (C_trz_) *	46.7	86.0
trz-Ar (N_trz_) *	47.5	71.1
trz–acac *	57.1	82.7

* Planes measured with the program Diamond 4.0.2; trz = triazolylidene.

## Supplementary Materials

Supplementary materials can be accessed at: http://www.mdpi.com/1420-3049/21/11/1561/s1.
