# Supplementary material for: Palladium(ii)-Acetylacetonato Complexes with Mesoionic Carbenes: Synthesis, Structures and Their Application in the Suzuki-Miyaura Cross Coupling Reaction
Source: Molecules. 2016 Nov 17;21(11):1561. doi: 10.3390/molecules21111561 (PMC6274386; doi:10.3390/molecules21111561)
Supplement: Supplementary file 1 [file molecules-21-01561-s001.pdf]

Supplementary Materials: Palladium(II)-Acetylacetonato Complexes with Mesoionic Carbenes: Synthesis, Structures and Their Application in the Suzuki-Miyaura Cross Coupling Reaction

Lara Hettmanczyk, Bianca Schmid, Stephan Hohloch and Biprajit Sarkar

Table of Contents

Crystallographic Data **S1**

Table S1. Parameters for the Data Collection and Structure Refinement for Complexes **1** and **3**.[1] ... S1

^{1}H- and ^{13}C-NMR spectroscopy **S2**

Figure S1. ^{1}H- (top) and ^{13}C-NMR (bottom) spectra of Complex **1** in $CDCl_3$ S2

Figure S2. ^{1}H- (top) and ^{13}C-NMR (bottom) spectra of Complex **2** in $CDCl_3$ S3

Figure S3. ^{1}H- (top) and ^{13}C-NMR (bottom) spectra of Complex **3** in $CDCl_3$ S4

Crystallographic Data

Table S1. Parameters for the Data Collection and Structure Refinement for Complexes **1** and **3**. [1]

	**1**	**3**
Chemical formula	$C_{23}H_{26}IN_3O_2Pd$	$C_{32}H_{44}IN_3O_2Pd$
M_r	609.77	736.00
Crystal system, space group	orthorombic, *Pcba*	monoclinic, $P2_1/n$
Temperature (K)	140(2)	140(2)
a, *b*, *c* (Å)	15.085(3), 15.908(4), 19.706(4)	10.241(5), 17.108(8), 18.463(9)
α, β, γ (°)	90.00, 90.00, 90.00	90, 99.587(9), 90
V (Å^3)	4728.9(18)	3190(2)
Z	8	4
Density (g/cm^3)	1.713	1.533
F000	2400	1488
Radiation type	Mo $\kappa\alpha$	Mo $\kappa\alpha$
μ (mm^{-1})	2.114	1.581
Crystal size (mm)	0.43 × 0.21 × 0.09	0.45 × 0.18 × 0.10
meas. refl.	36620	21058
indep. ref.	4153	7250
obsvd. [I > 2σ(I)] refl.	2328	5121
R_{int}	0.1795	0.0506
$R[F^2 > 2\sigma(F^2)]$, $wR(F^2)$, S	0.0603, 0.1732, 1.039	0.0480, 0.1253, 1.067
$\Delta\rho_{max}$, $\Delta\rho_{min}$ (e Å^{-3})	1.169, –1.515	2.230, –0.816
CCDC	965893	1015507

[1] Collected on a Bruker Smart AXS diffractometer using Mo $\kappa\alpha$ radiation (λ = 0.71073 Å).

^{1}H- and ^{13}C-NMR Spectroscopy

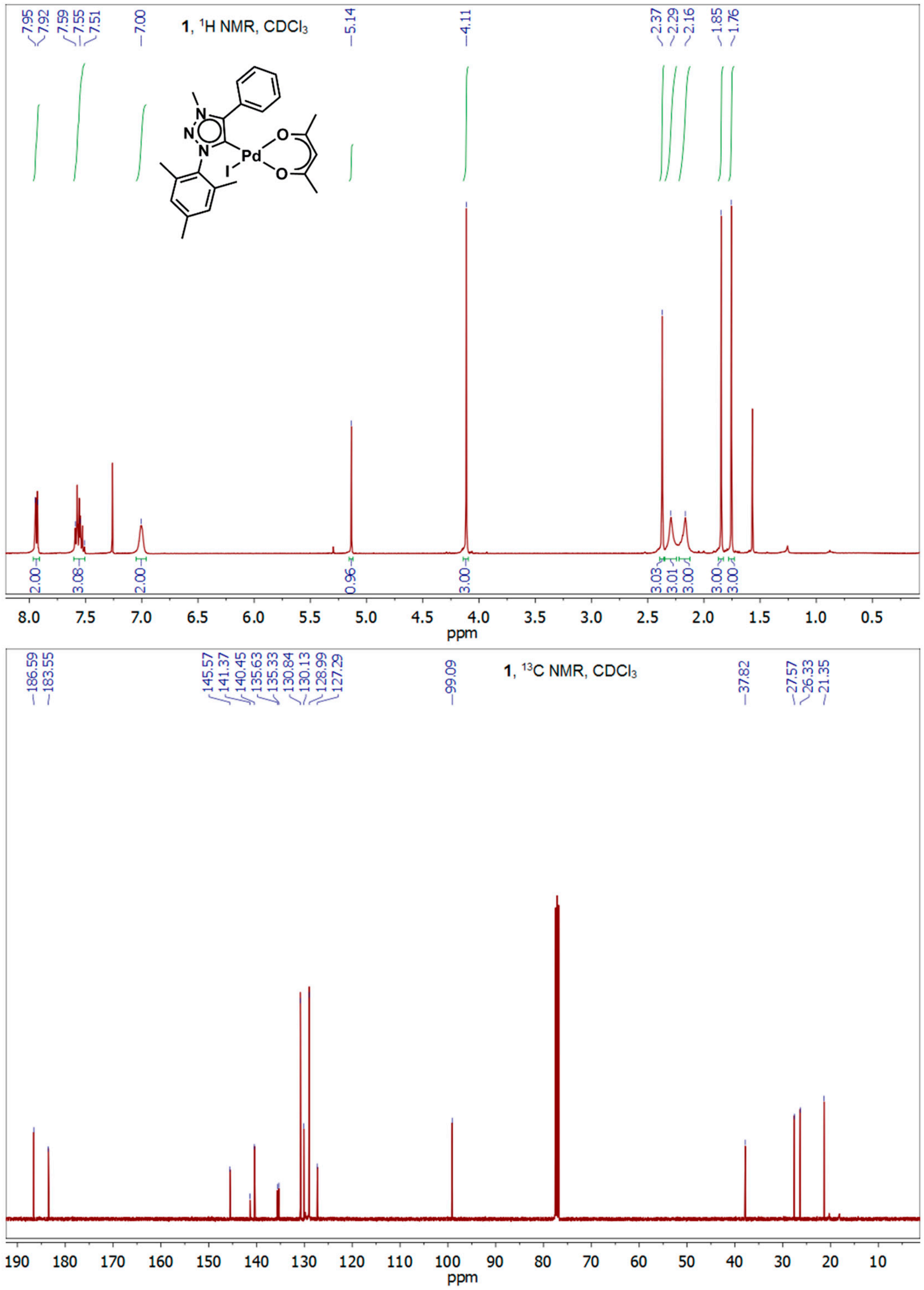

Figure S1. ^{1}H- (top) and ^{13}C-NMR (bottom) spectra of Complex **1** in $CDCl_3$.

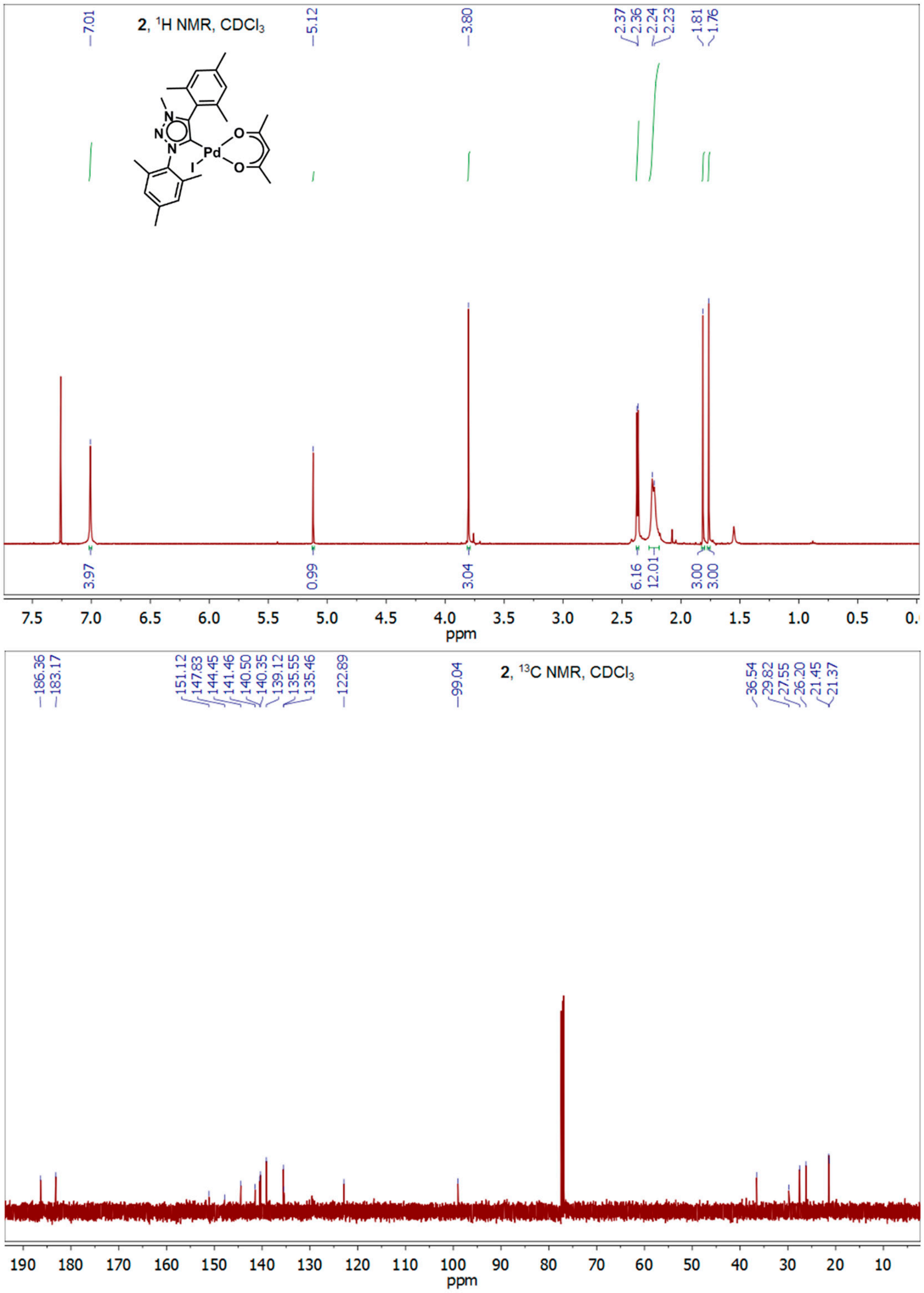

Figure S2. ^{1}H- (top) and ^{13}C-NMR (bottom) spectra of Complex **2** in $CDCl_3$.

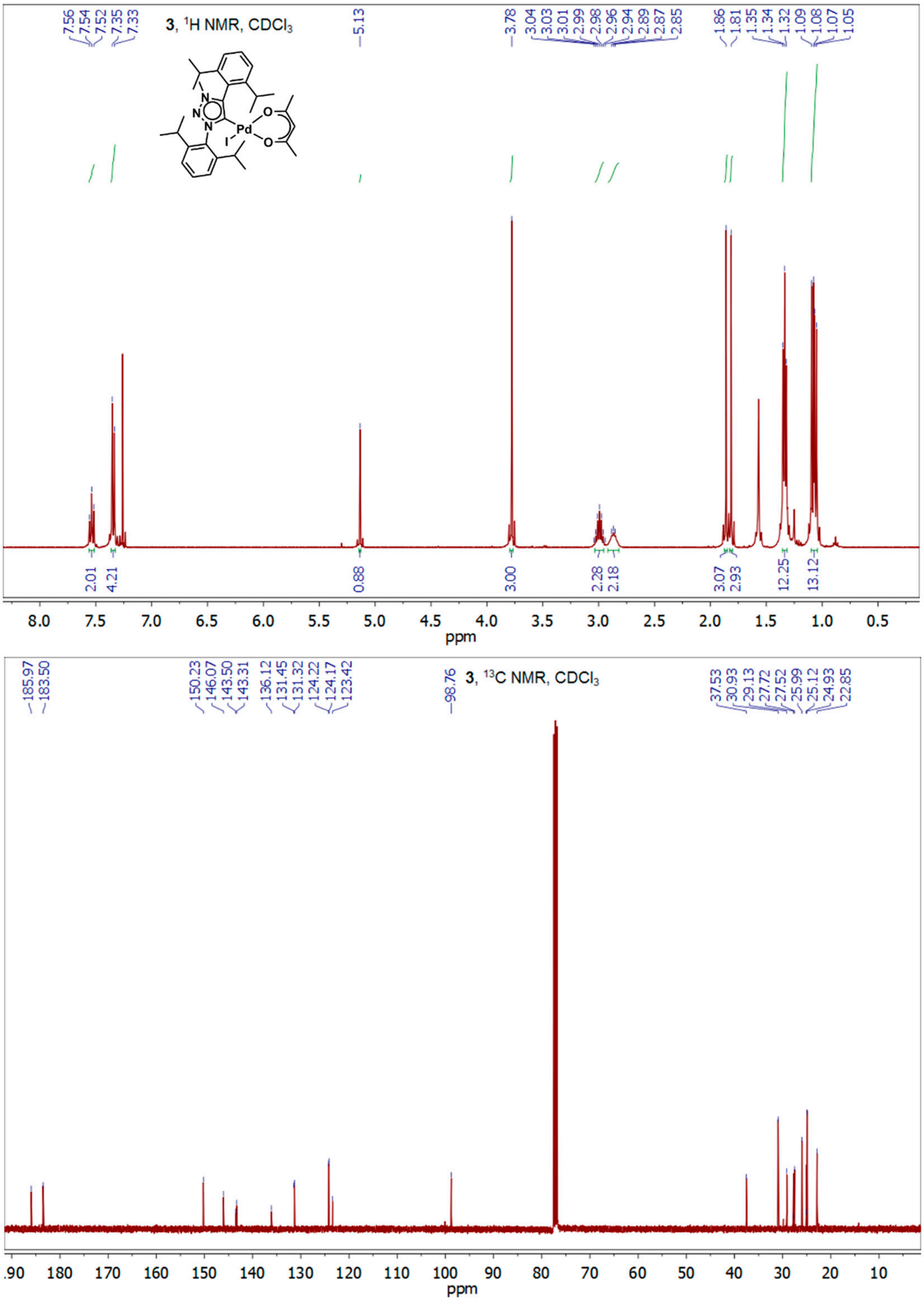

Figure S3. ^{1}H- (top) and ^{13}C-NMR (bottom) spectra of Complex **3** in $CDCl_3$.
